# The Development History and Research Tendency of Medical Informatics: Topic Evolution Analysis

**DOI:** 10.2196/31918

**Published:** 2022-01-27

**Authors:** Wenting Han, Xi Han, Sijia Zhou, Qinghua Zhu

**Affiliations:** 1 School of Management & Engineering Nanjing University Nanjing China; 2 School of Business Administration Guangdong University of Finance & Economics Guangzhou China; 3 Department of Information Systems City University of Hong Kong Hong Kong Hong Kong; 4 School of Information Management Nanjing University Nanjing China

**Keywords:** medical informatics, research hotspot, LDA model, topic evolution analysis, mobile phone

## Abstract

**Background:**

Medical informatics has attracted the attention of researchers worldwide. It is necessary to understand the development of its research hot spots as well as directions for future research.

**Objective:**

The aim of this study is to explore the evolution of medical informatics research topics by analyzing research articles published between 1964 and 2020.

**Methods:**

A total of 56,466 publications were collected from 27 representative medical informatics journals indexed by the Web of Science Core Collection. We identified the research stages based on the literature growth curve, extracted research topics using the latent Dirichlet allocation model, and analyzed topic evolution patterns by calculating the cosine similarity between topics from the adjacent stages.

**Results:**

The following three research stages were identified: early birth, early development, and rapid development. Medical informatics has entered the fast development stage, with literature growing exponentially. Research topics in medical informatics can be classified into the following two categories: data-centered studies and people-centered studies. Medical data analysis has been a research hot spot across all 3 stages, and the integration of emerging technologies into data analysis might be a future hot spot. Researchers have focused more on user needs in the last 2 stages. Another potential hot spot might be how to meet user needs and improve the usability of health tools.

**Conclusions:**

Our study provides a comprehensive understanding of research hot spots in medical informatics, as well as evolution patterns among them, which was helpful for researchers to grasp research trends and design their studies.

## Introduction

### Background

Medical informatics is a discipline that has received much attention in recent years. It has flourished with the development of information technology [1]. In 1959, Ledley and Lusted [2] suggested using computers to support medical decisions, which combined information technology with the medical domain. In the 1970s, the International Federation for Information Processing proposed the term *medical informatics*. It was defined as “the application of computer technology to all fields of medicine—medical care, medical teaching, and medical research.”

Systematic reviews of a research area are impactful because they can help researchers grasp future research trends and better design their studies. There have been many reviews of medical informatics conducted over the past 5 decades. Methods including bibliometric methods, visualization technologies, and social network analysis were always used in these reviews. For example, previous research used cocitation networks and co-occurring keywords to uncover knowledge structures in medical informatics [3], as well as keyword analysis [4] (such as keyword-frequency statistics and keyword clustering) to discover research topics. Visualization tools [5], including VOSviewer and CiteSpace, were used to reveal the scientific networks. In addition, some researchers brought MeSH (Medical Subject Headings) terms into medical informatics studies to extract high-quality research topics [6] or journals [7].

After reviewing medical informatics, we found that most systematic reviews in this field discovered research trends using bibliometric methods based on paper keywords, which summarized research contents into several words. Keywords, by contrast, had fewer semantic information compared with abstracts.

### Objectives

In this study, we chose the latent Dirichlet allocation (LDA) model to extract research topics from research article abstracts. Furthermore, we attempted to explore topic evolution patterns to predict future research trends. In conclusion, our study will be guided by the following three issues: (1) What are the research stages in the development of medical informatics, and what are the features of each stage? (2) What are the research hot spots in medical informatics and at different stages? Do these research hot spots change over time? (3) How have these research topics evolved over time? What will be the future research trends?

## Methods

### Data Collection

This study collected publications indexed by the Web of Science Core Collection database. To fully retrieve articles in medical informatics, we chose papers published by 27 representative medical informatics journals (Textbox 1) according to the medical informatics journal list supplied by the Journal Citation Reports. By limiting the document types into research articles and setting the published time before 2020, we downloaded the total records of 56,466 articles on April 16, 2021.

**Twenty-seven representative medical informatics journals (ranked by initials).**

**Titles of journals**

1. *Applied Clinical Informatics*
2. *Artificial Intelligence in Medicine*
3. *Biomedical Engineering—Biomedizinische Technik*
4. *BMC Medical Informatics and Decision Making*
5. *Cin—Computers Informatics Nursing*
6. *Computer Methods and Programs in Biomedicine*
7. *Health Informatics Journal*
8. *Health Information Management Journal*
9. *IEEE Journal of Biomedical and Health Informatics*
10. *Informatics for Health & Social Care*
11. *International Journal of Medical Informatics*
12. *International Journal of Technology Assessment in Health Care*
13. *Internet interventions—The Application of Information Technology in Mental and Behavioral Health*
14. *JMIR Medical Informatics*
15. *JMIR mHealth and uHealth*
16. *JMIR Serious Games*
17. *Journal of Biomedical Informatics*
18. *Journal of Evaluation in Clinical Practice*
19. *Journal of Medical Internet Research*
20. *Journal of Medical Systems*
21. *Journal of the American Medical Informatics Association*
22. *Medical & Biological Engineering & Computing*
23. *Medical Decision Making*
24. *Methods of Information in Medicine*
25. *Statistical Methods in Medical Research*
26. *Statistics in Medicine*
27. *Therapeutic Innovation & Regulatory Science*

### Research Design

#### Research Stage Identification

To determine how research topics evolve over time, we need to divide the history of medical informatics during the last 5 decades into several time units. Previous studies that analyzed publications released in the last 5-10 years usually took a year as a time unit [8]. When the time span exceeds decades, evidence for distinguishing time units, such as the life cycle theory [9], is necessary. In this study, we choose the literature growth curve of Price [10] to identify time units because this theory provides the quantitative features of literature growth in each stage. In the early stage, the number of research papers is minimal and increases unsteadily. At this point, no mathematical model perfectly fits the growth curve. Then, the number of research publications rises dramatically in the development stage, following the exponential increase model. In the mature stage, the number of papers grows slowly and steadily, with a growth trend that is consistent with the linear increase model. Finally, in the last stage of discipline, the number of papers declines as theories and research in 1 discipline become saturated. Furthermore, the growth curve would either gradually parallel the horizontal axis or fluctuate irregularly.

According to the literature growth curve of Price [10], a discipline’s development history can be divided into stages based on the rate of literature growth. To divide the past 5 decades of medical informatics into distinct stages, we used the piecewise regression algorithm to fit the curve of the annual cumulative number of research papers. The time point that can separate the development stages occurs when the curve slopes are significantly distinguished. After identifying these time points, we attempted to match the literature growth curve in every stage with various mathematical models (linear increase model, exponential increase model, etc) to find the features of each stage.

#### Topic Evolution Analysis

Topic evolution analysis was adopted in this study to extract research topics and explore their evolution patterns. There are many topic extraction methods, including those based on word frequency, co-occurrence, and topic models. Compared with the first 2 methods, extracting topics through topic models, which can mine topics from a semantic perspective and show a better topic distribution, is suitable for our research. From various topic models, we chose the LDA model [11] for topic extraction. The LDA model uses the Dirichlet distribution to perform probability modeling at three levels: document, topic, and word. It calculates the semantic similarities between topics, documents, topics, and keywords. Many previous studies have shown that this model is effective in research topic mining and research trend prediction [12,13]. Before extracting topics using the LDA model, we had to determine the optimal number of topics extracted. Perplexity [11] and coherence [14] are always chosen as indicators. The optimal number of topics occurs when the value of perplexity is low, and the value of coherence is high.

**Figure 1:**
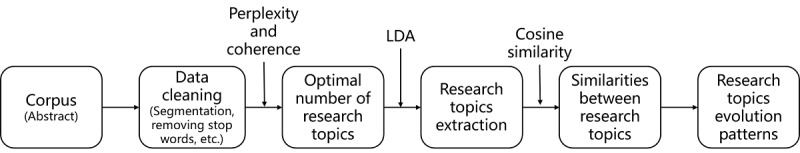
The process of topic evolution analysis. LDA: latent Dirichlet allocation.

Then, we needed to calculate the similarity between topics from adjacent stages to identify their relationships. Previous studies have used semantic similarity between keywords under 2 topics to represent topic similarity [15,16]. If the similarity of 2 keyword vectors exceeds a threshold, the evolutionary relationship between 2 topics is identified; otherwise, it is not. Typical measures of word vector similarity include Jensen-Shannon divergence, Kullback-Leibler divergences, and cosine similarity [16,17]. In this study, we used Python coding programs to calculate the cosine similarity between the 2 topics. The cosine similarity value ranges from 0 to 1, with higher values indicating greater similarity. It is reasonable to take 0.5 as a threshold. Figure 1 provides an overview of the topic evolution analysis process.

## Results

### Identify Research Stages

**Figure 2:**
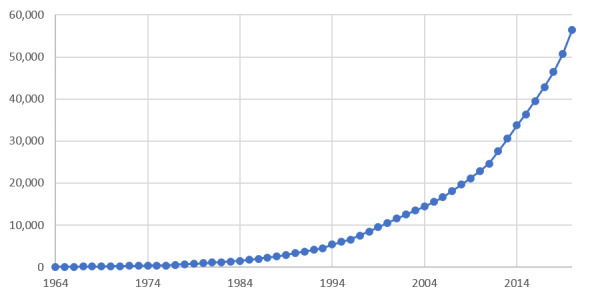
Annual distribution of the cumulative number of research papers.

As stated previously, we counted the annual cumulative number of research papers and plotted the literature growth curves in Figure 2.

**Figure 3:**
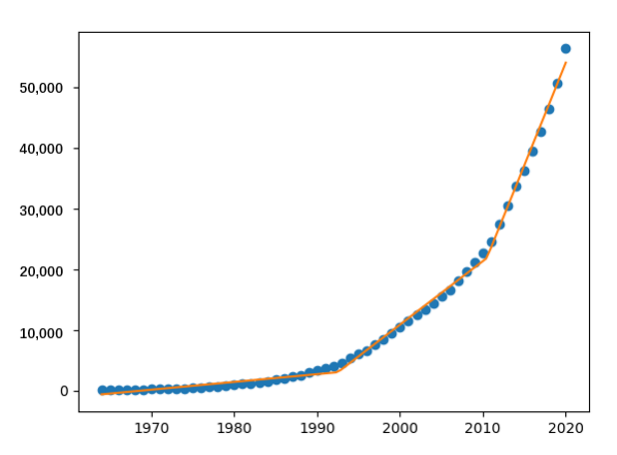
The result of piecewise regression fitting.

Then, to find the points that significantly distinguish the rate of literature growth, we used the piecewise regression algorithm in Python to fit the curve of the annual cumulative number of papers in Figure 2. The fitting results are shown in Figure 3.

Figure 3 indicates that the curve was inflected in 1992 and 2010. Therefore, we divided the past 5 decades into three stages: 1964-1991, 1992-2009, and 2010-2020. We then adopted SPSS (IBM Corporation) to fit the growth curve for each stage. Curve fitting yielded the following results.

**Figure 4:**
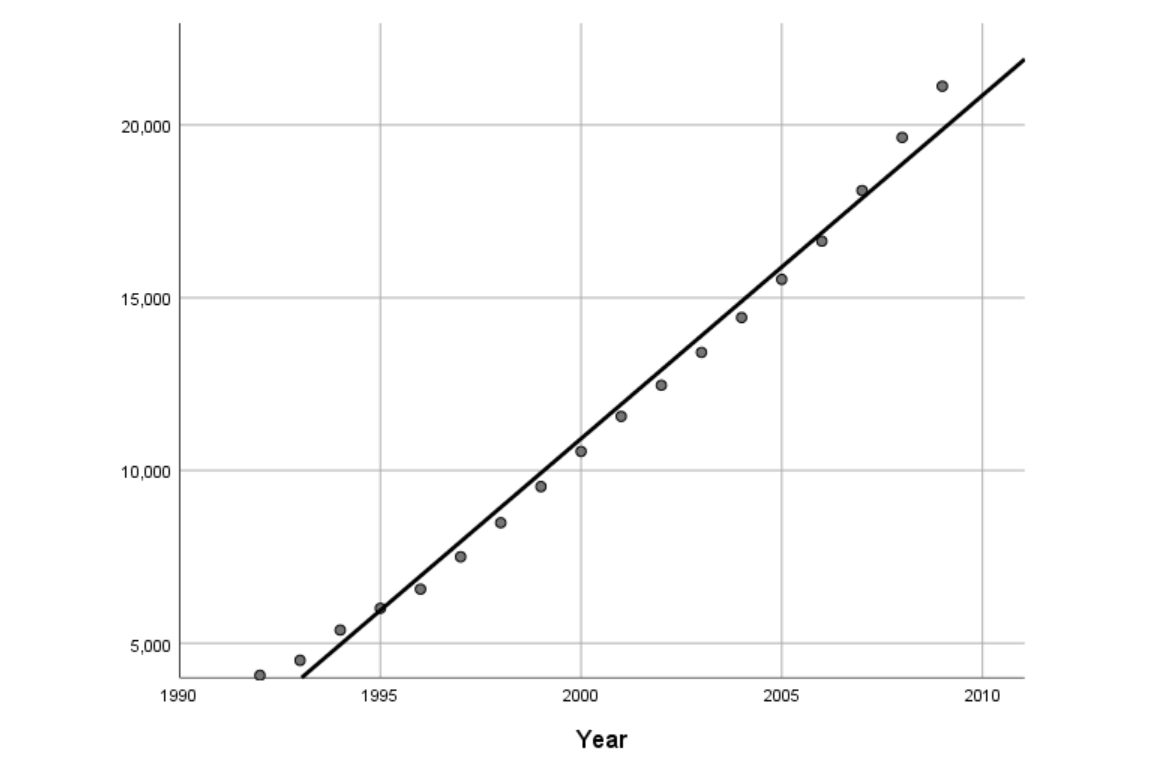
Results of curve fitting (1992-2009).

**Figure 5:**
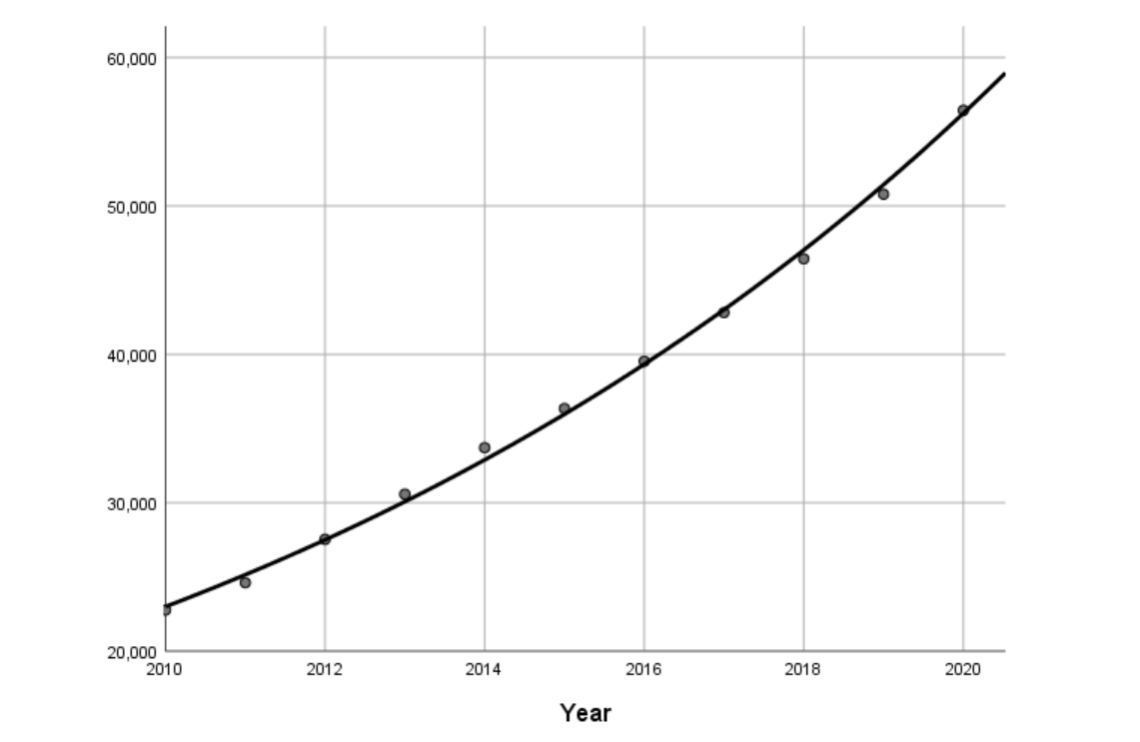
Results of curve fitting (2010-2020).

The literature growth curve between 1964 and 1991 was difficult to fit any mathematical models. The literature growth curve from 1992 to 2009 (Figure 4) was consistent with the linear increase model, and the adjusted *R*^2^ was 0.988. The literature growth curve from 2010 to 2020 (Figure 5) followed the exponential increase model, and the adjusted *R*^2^ was 0.998. Then, we can summarize the 3 stages of medical informatics: the period from 1964 to 1991 belonged to the early birth stage of medical informatics. There were fewer papers at this point, and the rising speed was unstable. The period of 1992-2009 could be regarded as the early development stage, as the number of papers began to increase and the rate of growth fitted a linear increase model but had not yet reached an exponential increase. Finally, between 2010 and 2020, medical informatics came to a rapid development stage. Some emerging technologies, such as deep learning algorithms and open-source tools for artificial intelligence, have been released and boomed up with the big data era. How to use these technologies in medical informatics has been widely discussed. Therefore, the number of publications increased significantly, and the growth curve followed the exponential increase model.

### Topic Evolution Analysis

#### Overview

We used the LDA model to extract research topics from all corpora and corpora of each stage. As mentioned above, the abstracts of the research articles were chosen as corpora because the abstract, as a paragraph of text, had a clearer semantic logic and a more complete summary of the paper’s content, making it more appropriate for LDA-based research topic extraction.

#### Optimal Topic Number Identification

**Figure 6:**
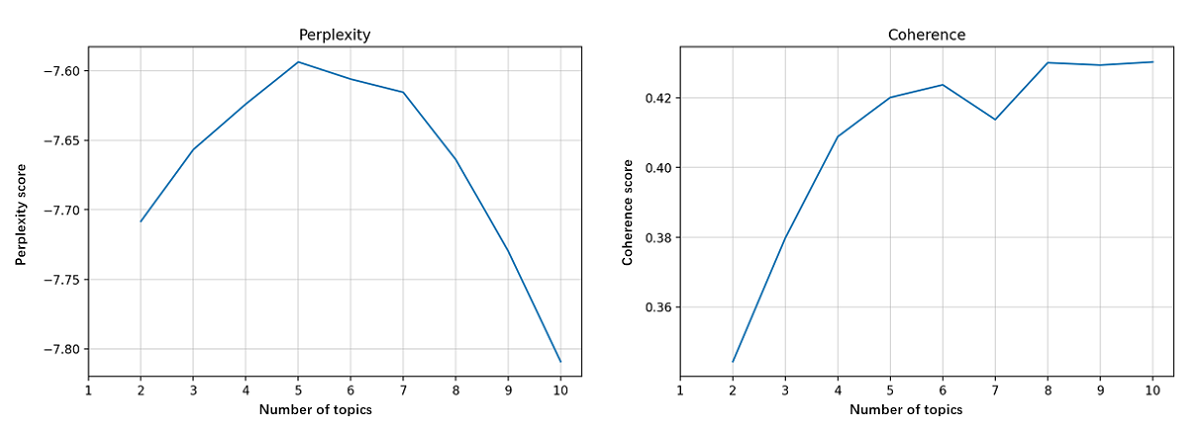
The perplexity and coherence curve of all corpora.

**Figure 7:**
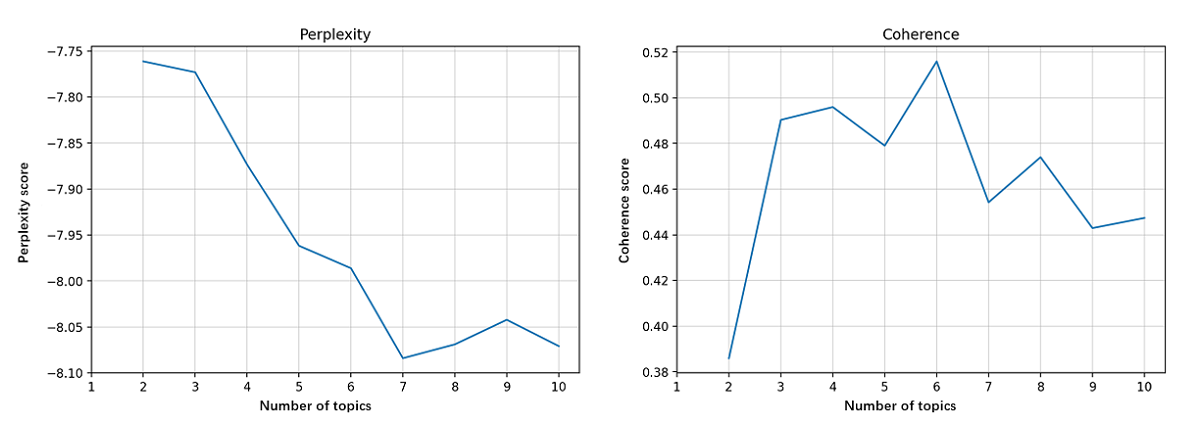
The perplexity and coherence curve of corpora in research stage 1 (1964-1991).

**Figure 8:**
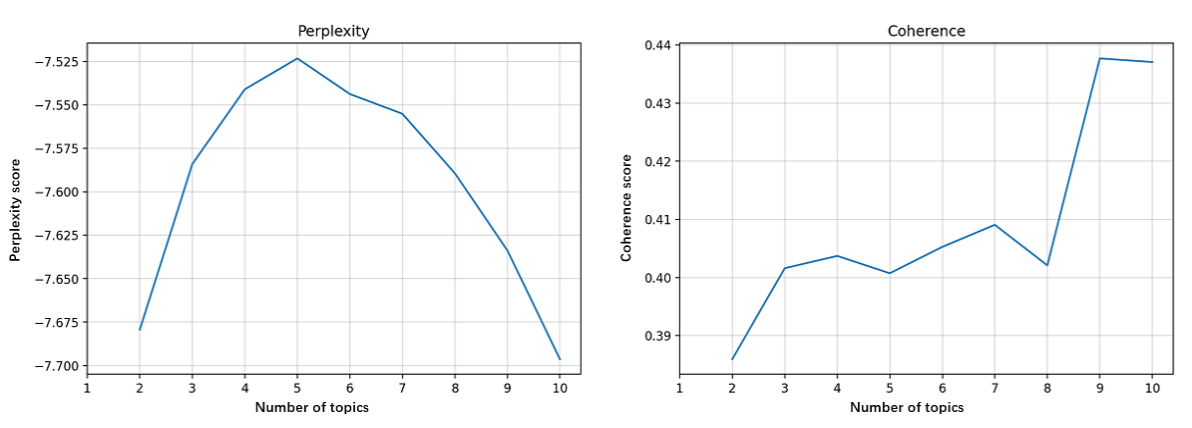
The perplexity and coherence curve of corpora in research stage 2 (1992-2009).

**Figure 9:**
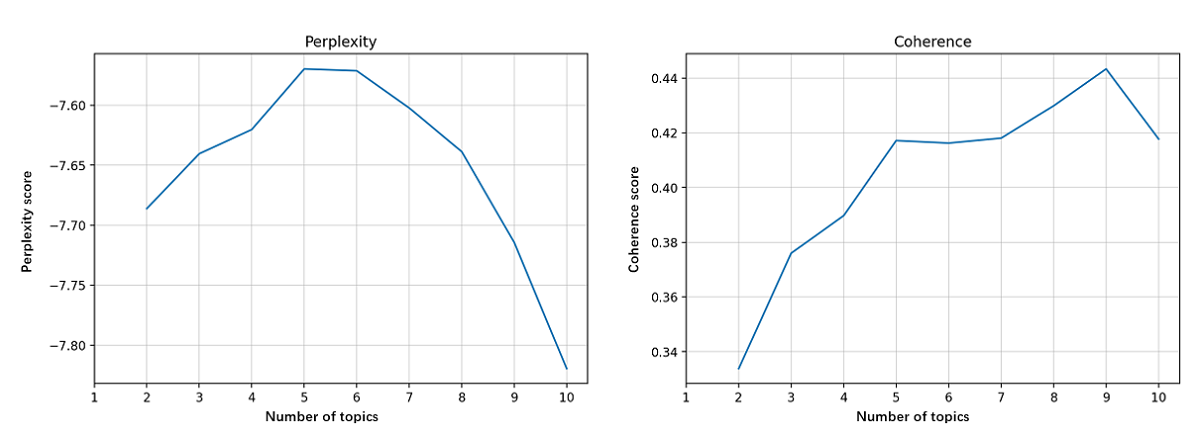
The perplexity and coherence curve of corpora in research stage 3 (2010-2020).

Perplexity and coherence were calculated to identify the optimal number of topics extracted. Figures 6-9 show the perplexity and coherence curves drawn by Python coding programs.

Perplexity is an index that measures the information generalized by the topic model. A lower perplexity value indicates that the topic model provides more information. Coherence measures the degree of semantic similarity between keywords within a topic. Because topics learned by topic models are not always fully interpretable, coherence is proposed to distinguish between interpretable and artificial topics [14]. A higher coherence score indicates that the topic model offers some meaningful topics. We need to balance perplexity and coherence to choose the optimum number of topics with lower perplexity and higher coherence. We also proposed that higher coherence was more significant because we tended to get more relevant topics.

Figure 6 shows that the optimum number of topics in all corpora was 10, with maximum coherence and minimum perplexity. Figure 7 shows that the coherence reached its maximum when the number of topics was 6, whereas the perplexity was lowest for 7 topics. However, we determined to extract 6 topics from the corpora of stage 1. As seen in Figures 8 and 9, the coherence curve reached the end of the rapid growth when the number of topics was 9. Meanwhile, perplexity was relatively low at 9 topics. We then decided to extract 9 topics from the corpora of stages 2 and 3.

#### Research Topic Extraction

We adopted the LDA model to extract research topics from the abstracts of 56,466 research articles. The Python library Gensim was used to conduct the LDA model. Gensim is a Python library for topic modeling, document indexing, and similarity retrieval with large corpora. *Alpha* and *beta* are hyperparameters that affect topics’ sparsity. According to the Gensim docs, they both default to 1.0/number of topics prior. The number of topics extracted was set to 10, and the top 20 keywords were displayed under each topic. The topic extraction results for all corpora are shown in Table 1.

Table 1 provides an overview of the 10 research hot spots in medical informatics. Topic 1 focused primarily on the medical system, and the keywords under this topic indicate that development and usage, medical system technology, and users’ needs are all explored. Topic 2 mainly concerned health-related measurement, with researchers focusing on developing health domain scales, for example, health literacy. Questionnaire design and item optimization are important research questions on this topic. Topic 3 was related to patient care. Physicians, clinicians, treatment, and risk become the top keywords with high weights in this topic. Studies under topic 4 were largely concerned with web-based health information, including the search, use, and evaluation of web-based health information. In addition, user profiling and participation in web-based health communities are hot spots under this topic. Topic 5 can be summarized as medical image processing. Under this topic, researchers were interested in the development and optimization of image-processing algorithms. The keywords under topic 6 were mostly connected to health data analysis. The use of mathematical models and information technologies, such as simulations, in health data analysis has attracted many researchers. Topic 7 was primarily concerned with medication management. Researchers have emphasized drug prescription, dose, safety, and surveillance. Topic 8 emphasized the studies on electronic medical records, especially the management, analysis, and application of medical records. The major research content under topic 9 concerned health interventions. The experimental method is commonly used in health intervention studies. Participants were recruited and divided into groups with different types of interventions. The efficacy of interventions was verified by comparing the performances of various groups. Finally, topic 10 was mainly concerned with the analysis of physiological data collected from patients, such as electroencephalography and heart rate. Relevant keywords include signal, frequency, and flow rate.

We also extracted research hot spots at each stage. Table 2 shows the research topics and top keywords in the 3 stages.

The keywords in stage 1 indicated that the research topics were more medically connected, with a concentration on medical data analysis. For example, topic 4 mainly focused on the analysis of patients’ physiological data (blood, flow, signal, arterial, etc). The analysis and application of data in medical systems was the focus of topic 5 (system, datum, program, etc). Meanwhile, researchers were interested in learning how to analyze the aforementioned medical data. Model, variable, estimation, linear, and other keywords in topic 6 suggested that mathematical models and computational techniques were effective methods in medical data analysis.

Stages 1 and 2 covered some comparable topics, with topics 2 and 4 in stage 2 maintaining the focus on medical system development and medical data analysis methods. Meanwhile, topics in stage 2 revealed some new patterns. For example, the types of medical data were enlarged in the focus of medical data analysis, with medical image processing emerging as a new research hot spot (topic 3 in stage 2). Furthermore, topic 5 suggested that researchers were concerned about the search, application, and users’ need for web-based health information. Furthermore, topics in stage 2 revealed that the attention on patients began to increase, such as topic 1, which focused on patient care and treatment, and topic 8, which addressed patients’ need to improve medical institutions’ services.

Topics in stage 3 inherited the focus on medical data analysis from stage 1 and stage 2, including analysis of medical system data (topic 1), methods of medical data analysis (topic 2), analysis of patients’ electronic medical records (topic 3), medical image processing (topic 8), and analysis of disease-related data (topic 9). The keywords in these topics indicated that the goal of medical data analysis is gradually shifting to human-centered, such as improving medical systems based on patients’ needs (topic 1), providing better care for patients (topic 3), identifying health risks, and predicting disease for patients (topic 9).

There were a few new topics in stage 3. First, it is worth noting that the development of health tools has become a research hot spot. Topic 4 showed how health tools, such as sensing devices, were used to collect users’ physiological data and help them with self-health management. Furthermore, mobile health tools were used for health interventions (topic 5). Meanwhile, as seen in topic 7, which addressed the measurement of health tool usability, user experience has been one of the research hot spots in medical informatics. Finally, the researchers emphasized the importance of standard medical information. The keywords in topic 6 revealed that the construction of concepts, terms, and ontologies became a popular topic in stage 3.

**Table 1:** Research topics and top keywords in all corpora.

Topics	Keywords
Topic 1	0.026*system, 0.014*information, 0.014*health, 0.012*datum, 0.011*medical, 0.010*care, 0.009*technology, 0.008*process, 0.008*base, 0.008*clinical, 0.007*support, 0.007*user, 0.007*provide, 0.007*use, 0.007*develop, 0.007*development, 0.006*application, 0.006*need, 0.005*implementation, 0.005*tool
Topic 2	0.017*score, 0.016*use, 0.015*measure, 0.013*student, 0.011*scale, 0.010*assess, 0.009*quality, 0.009*test, 0.009*assessment, 0.008*health, 0.008*questionnaire, 0.008*age, 0.008*item, 0.008*group, 0.008*high, 0.007*factor, 0.007*difference, 0.007*mean, 0.006*level, 0.006*year
Topic 3	0.069*patient, 0.020*care, 0.019*cost, 0.017*decision, 0.016*physician, 0.015*treatment, 0.014*clinical, 0.010*practice, 0.008*guideline, 0.008*evidence, 0.007*effectiveness, 0.006*use, 0.006*outcome, 0.006*decision_make, 0.006*benefit, 0.005*clinician, 0.005*primary, 0.005*quality, 0.005*year, 0.005*risk
Topic 4	0.047*health, 0.031*information, 0.013*use, 0.013*internet, 0.011*online, 0.011*search, 0.010*survey, 0.008*relate, 0.007*web, 0.007*user, 0.007*access, 0.006*public, 0.006*identify, 0.006*age, 0.006*population, 0.005*question, 0.005*community, 0.005*source, 0.005*report, 0.005*woman
Topic 5	0.018*image, 0.017*use, 0.014*base, 0.013*propose, 0.012*feature, 0.010*classification, 0.008*performance, 0.008*datum, 0.007*model, 0.007*accuracy, 0.007*algorithm, 0.007*system, 0.006*technique, 0.006*set, 0.006*analysis, 0.006*network, 0.005*detection, 0.005*different, 0.005*show, 0.005*present
Topic 6	0.030*model, 0.017*datum, 0.014*use, 0.013*estimate, 0.011*effect, 0.011*analysis, 0.011*test, 0.010*trial, 0.009*time, 0.008*propose, 0.008*study, 0.007*treatment, 0.007*base, 0.006*simulation, 0.006*distribution, 0.005*parameter, 0.005*compare, 0.005*case, 0.005*outcome, 0.005*variable
Topic 7	0.043*drug, 0.021*medication, 0.013*order, 0.013*dose, 0.012*alert, 0.011*error, 0.011*safety, 0.009*rate, 0.009*report, 0.008*prescription, 0.008*system, 0.008*infection, 0.007*increase, 0.007*surveillance, 0.007*time, 0.007*period, 0.006*use, 0.006*event, 0.006*prescribe, 0.006*identify
Topic 8	0.029*patient, 0.024*datum, 0.018*clinical, 0.018*use, 0.012*record, 0.012*system, 0.011*model, 0.010*hospital, 0.009*medical, 0.007*identify, 0.007*disease, 0.006*base, 0.006*time, 0.006*concept, 0.006*develop, 0.006*database, 0.006*report, 0.006*set, 0.006*code, 0.005*diagnosis
Topic 9	0.026*intervention, 0.020*group, 0.019*participant, 0.011*base, 0.011*app, 0.010*program, 0.010*self, 0.008*use, 0.008*month, 0.007*control, 0.007*health, 0.007*change, 0.007*week, 0.006*behavior, 0.006*follow, 0.006*time, 0.006*user, 0.006*increase, 0.005*day, 0.005*mobile
Topic 10	0.014*use, 0.011*signal, 0.008*model, 0.008*time, 0.007*system, 0.006*measurement, 0.006*parameter, 0.006*frequency, 0.005*show, 0.005*change, 0.005*flow, 0.005*rate, 0.005*subject, 0.005*high, 0.004*analysis, 0.004*heart, 0.004*control, 0.004*increase, 0.004*different, 0.004*measure

**Table 2:** Research topics and keywords in the 3 stages.

Topics		Keywords
**Stage 1 (1964-1991)**		
	Topic 1	0.022*patient, 0.008*subject, 0.008*risk, 0.008*use, 0.007*record, 0.007*analysis, 0.006*image, 0.006*dose, 0.005*power, 0.005*procedure, 0.005*disease, 0.005*number, 0.005*measure, 0.005*system, 0.005*calculate, 0.005*datum, 0.005*average, 0.005*step, 0.005*present, 0.005*base
	Topic 2	0.018*provide, 0.015*medical, 0.015*increase, 0.015*physician, 0.013*include, 0.010*several, 0.009*use, 0.008*year, 0.008*report, 0.008*risk, 0.008*practice, 0.007*patient, 0.007*model, 0.007*analysis, 0.007*probability, 0.006*condition, 0.006*factor, 0.006*investigate, 0.006*value, 0.006*heart
	Topic 3	0.015*test, 0.014*clinical, 0.012*trial, 0.012*estimate, 0.011*analysis, 0.010*model, 0.009*diagnostic, 0.008*treatment, 0.008*compare, 0.008*medical, 0.008*problem, 0.008*rate, 0.007*decision, 0.007*present, 0.006*population, 0.006*development, 0.006*need, 0.006*effect, 0.006*statistical, 0.006*multiple
	Topic 4	0.017*blood, 0.017*measurement, 0.016*flow, 0.016*model, 0.015*analysis, 0.014*pressure, 0.013*use, 0.011*electrode, 0.010*signal, 0.008*measure, 0.008*human, 0.007*effect, 0.006*impedance, 0.006*spectral, 0.006*arterial, 0.006*volume, 0.005*parameter, 0.005*frequency, 0.005*distribution, 0.005*skin
	Topic 5	0.042*system, 0.027*datum, 0.013*clinical, 0.012*information, 0.011*program, 0.011*use, 0.011*medical, 0.011*knowledge, 0.010*computer, 0.010*base, 0.009*analysis, 0.009*image, 0.009*develop, 0.007*management, 0.007*describe, 0.007*process, 0.006*processing, 0.006*trial, 0.006*procedure, 0.005*study
	Topic 6	0.019*use, 0.019*model, 0.010*time, 0.009*study, 0.008*control, 0.008*propose, 0.008*variable, 0.008*individual, 0.008*analysis, 0.007*first, 0.006*describe, 0.006*datum, 0.006*make, 0.006*number, 0.006*non, 0.005*estimation, 0.005*response, 0.005*linear, 0.005*examine, 0.005*base
**Stage 2 (1992-2009)**		
	Topic 1	0.032*patient, 0.018*use, 0.012*diagnosis, 0.011*diagnostic, 0.011*datum, 0.010*classification, 0.009*test, 0.009*case, 0.009*accuracy, 0.008*performance, 0.008*model, 0.008*hospital, 0.007*sensitivity, 0.007*clinical, 0.007*system, 0.007*set, 0.006*compare, 0.006*disease, 0.006*rate, 0.006*prediction
	Topic 2	0.027*system, 0.018*datum, 0.015*medical, 0.012*information, 0.012*base, 0.011*use, 0.011*clinical, 0.010*model, 0.009*knowledge, 0.008*application, 0.007*develop, 0.007*describe, 0.007*support, 0.006*provide, 0.006*process, 0.006*concept, 0.005* software, 0.005*present, 0.005*database, 0.005*tool
	Topic 3	0.018*image, 0.017*use, 0.013*signal, 0.008*time, 0.008*analysis, 0.007*base, 0.007*system, 0.007*technique, 0.006*frequency, 0.005*obtain, 0.005*show, 0.005*present, 0.005*feature, 0.005*high, 0.005*measurement, 0.005*subject, 0.004*parameter, 0.004*noise, 0.004*different, 0.004*measure
	Topic 4	0.025*model, 0.016*datum, 0.014*use, 0.014*test, 0.011*analysis, 0.011*estimate, 0.011*effect, 0.010*trial, 0.008*propose, 0.007*treatment, 0.007*time, 0.007*base, 0.007*study, 0.006*distribution, 0.006*parameter, 0.005*compare, 0.005*simulation, 0.005*error, 0.005*clinical, 0.005*procedure
	Topic 5	0.018*information, 0.011*use, 0.009*health, 0.009*evidence, 0.009*report, 0.009*search, 0.008*user, 0.008*clinical, 0.008*internet, 0.007*evaluation, 0.007*identify, 0.007*base, 0.006*assessment, 0.006*question, 0.006*study, 0.006*technology, 0.005*web, 0.005*quality, 0.005*medical, 0.005*provide
	Topic 6	0.018*model, 0.011*cell, 0.009*use, 0.008*gene, 0.008*dose, 0.007*tissue, 0.007*increase, 0.006*flow, 0.006*pressure, 0.006*blood, 0.006*change, 0.005*drug, 0.005*control, 0.005*measure, 0.004*show, 0.004*current, 0.004*high, 0.004*response, 0.004*experimental, 0.004*value
	Topic 7	0.028*patient, 0.018*cost, 0.014*treatment, 0.011*health, 0.010*use, 0.009*group, 0.008*risk, 0.007*measure, 0.007*intervention, 0.007*decision, 0.007*year, 0.006*analysis, 0.006*quality, 0.006*compare, 0.006*score, 0.006*high, 0.006*utility, 0.006*outcome, 0.005*life, 0.005*state
	Topic 8	0.026*health, 0.024*care, 0.020*patient, 0.015*system, 0.013*information, 0.009*medical, 0.009*practice, 0.008*physician, 0.008*hospital, 0.008*technology, 0.007*service, 0.007*clinical, 0.006*base, 0.006*computer, 0.006*use, 0.005*need, 0.005*management, 0.005*record, 0.005*support, 0.004*implementation
	Topic 9	0.021*model, 0.021*disease, 0.019*risk, 0.017*datum, 0.015*estimate, 0.013*time, 0.011*population, 0.011*age, 0.011*rate, 0.010*use, 0.009*exposure, 0.009*case, 0.008*incidence, 0.008*cancer, 0.007*infection, 0.007*child, 0.007*mortality, 0.006*year, 0.005*individual, 0.005*prevalence
**Stage 3 (2010-2020)**		
	Topic 1	0.023*health, 0.018*datum, 0.015*information, 0.012*system, 0.008*technology, 0.008*clinical, 0.008*medical, 0.007*process, 0.007*provide, 0.007*use, 0.007*support, 0.007*care, 0.006*base, 0.006*need, 0.006*development, 0.005*develop, 0.005*service, 0.005*patient, 0.005*user, 0.005*healthcare
	Topic 2	0.031*model, 0.016*datum, 0.014*use, 0.012*effect, 0.011*estimate, 0.011*treatment, 0.010*trial, 0.010*analysis, 0.009*time, 0.008*propose, 0.008*study, 0.008*test, 0.007*outcome, 0.006*base, 0.006*simulation, 0.005*compare, 0.005*risk, 0.004*clinical, 0.004*show, 0.004*variable
	Topic 3	0.056*patient, 0.024*care, 0.016*system, 0.016*hospital, 0.012*physician, 0.010*use, 0.009*clinical, 0.009*electronic, 0.009*medication, 0.009*record, 0.008*time, 0.008*information, 0.008*health, 0.007*medical, 0.006*ehr, 0.006*provider, 0.006*improve, 0.006*practice, 0.006*quality, 0.005*decision
	Topic 4	0.013*use, 0.009*model, 0.007*time, 0.007*patient, 0.007*system, 0.006*measurement, 0.006*parameter, 0.006*subject, 0.006*show, 0.005*control, 0.005*rate, 0.005*change, 0.005*device, 0.005*measure, 0.005*sensor, 0.005*high, 0.004*activity, 0.004*signal, 0.004*heart, 0.004*increase
	Topic 5	0.020*health, 0.017*intervention, 0.015*participant, 0.013*group, 0.010*use, 0.010*app, 0.009*base, 0.007*self, 0.007*online, 0.007*internet, 0.006*user, 0.006*conclusion, 0.005*report, 0.005*web, 0.005*behavior, 0.005*high, 0.005*program, 0.005*information, 0.005*increase, 0.005*treatment
	Topic 6	0.012*system, 0.012*model, 0.011*use, 0.011*clinical, 0.011*concept, 0.008*base, 0.007*term, 0.007*medical, 0.007*text, 0.007*semantic, 0.007*ontology, 0.007*biomedical, 0.007*knowledge, 0.006*information, 0.006*cell, 0.006*structure, 0.006*domain, 0.006*query, 0.005*different, 0.005*document
	Topic 7	0.016*use, 0.013*score, 0.013*usability, 0.012*student, 0.011*test, 0.010*base, 0.010*user, 0.009*item, 0.009*evaluation, 0.009*training, 0.008*tool, 0.008*assessment, 0.008*group, 0.008*assess, 0.008*evaluate, 0.008*questionnaire, 0.007*develop, 0.007*scale, 0.007*nursing, 0.007*task
	Topic 8	0.019*propose, 0.019*use, 0.018*image, 0.015*feature, 0.014*base, 0.011*classification, 0.010*performance, 0.009*accuracy, 0.007*datum, 0.007*detection, 0.007*algorithm, 0.007*model, 0.006*technique, 0.006*system, 0.006*signal, 0.006*show, 0.005*analysis, 0.005*classifier, 0.005*high, 0.005*dataset
	Topic 9	0.021*datum, 0.020*disease, 0.017*use, 0.015*drug, 0.014*identify, 0.011*cancer, 0.011*risk, 0.010*clinical, 0.010*patient, 0.008*base, 0.008*gene, 0.007*diagnosis, 0.007*develop, 0.007*set, 0.006*predict, 0.006*case, 0.006*high, 0.006*prediction, 0.005*record, 0.005*accuracy

#### Topic Evolution Pattern Construction

As previously stated, there were several research topics that were comparable between 2 adjacent stages. To determine the evolution pattern, we used the Python coding program to calculate the cosine similarity of keywords between 2 research topics from 2 adjacent stages. A total of 2 topics were connected if the cosine similarity between them was more than 0.5. Figure 6 illustrates the connections between topics from stages 1 to 3. Here, S1-T5 refers to topic 5 in stage 1.

**Figure 10:**
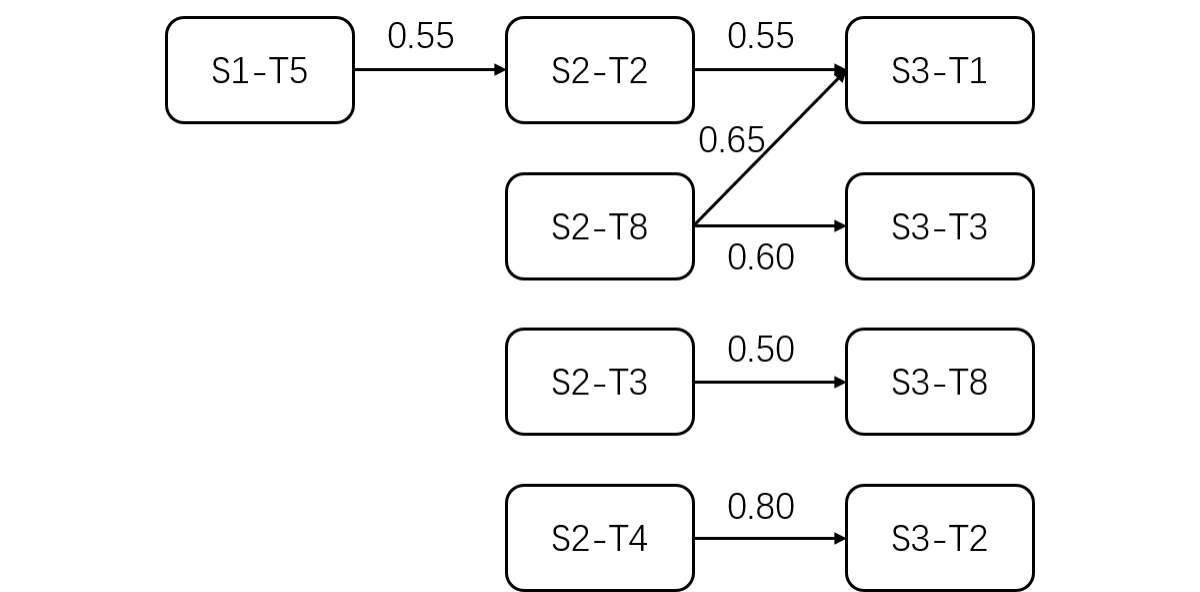
Research topics’ evolution patterns.

Figure 10 shows that the connections between stage 1 and stage 2 were weaker than those between stage 2 and stage 3. The reason for this could be that, in the early stage of medical informatics, there was less research literature and the focus of these studies was primarily on the medical field, whereas as medical informatics developed, research became more interdisciplinary as knowledge and research methods from other fields, such as computer science, library science, and psychology, were integrated into medical informatics. Therefore, research topics in stages 2 and 3 were more diverse and less similar to those in stage 1.

There was an evolution line from stage 1 to stage 3, starting at topic 5 in stage 1, moving through topic 2 in stage 2, and ending at topic 1 in stage 3. The focus of these topics was mainly on medical systems, with the difference that topic 5 in stage 1 and topic 2 in stage 2 concentrated more on technologies for medical system development and optimization, such as software and database construction, whereas topic 1 in stage 3 addressed the user needs to improve the service of the medical system.

There were several evolution lines between topics in stages 2 and 3. First, topic 8 in stage 2 was split into topic 1 and topic 3 in stage 3. The keywords of topic 8 in stage 2 emphasized the importance of patient needs. As a result, topic 1 in stage 3 evaluated patient needs in the progress of medical system development, and ’topic 3 in stage 3 considered patient needs in the improvement of health care service. Second, topic 8 in stage 3 was inherited from topic 3 in stage 2, indicating that medical image processing has been one of the research hot spots in medical informatics since the 1990s. Finally, topic 4 in stage 2 evolved into topic 2 in stage 3, with the focus of this evolution line being primarily on methods of medical data analysis. Researchers have been working hard to develop efficient methods for analyzing medical data, such as using mathematical models and constructing computing algorithms.

## Discussion

### Principal Findings

This study explored the research stages, research hot spots, and their evolution patterns in medical informatics. We found that medical informatics has gone through three stages: (1) the early birth stage (1964-1991), with a small number of papers and an unstable growth speed; (2) the early development stage (1992-2009), with an increasing number of papers and a steadily rising speed; and (3) the fast development stage (2010-2020), with a large number of papers and an exponential growth speed.

In the first stage (1964-1991), researchers focused on medical data analysis, including the analysis of patients’ physiological data, such as pulmonary data [18], cerebrum data [19], and renal data [20], as well as the analysis of medical images, such as electroencephalogram [21] and electromyography [22]. Medical data analysis studies in this period served a primary role in in the field of medicine, such as providing therapy for patients or assisting physicians with disease diagnosis. In addition, methodologies and technologies used in medical data analysis became a research hot spot in this period. Researchers used some mathematical models (regression [23], Bayesian [24,25], Markov [26], etc) and computer technologies (database [27], information system [28], simulation [29], etc) to improve the efficiency and precision of medical data analysis.

In the second stage (1992-2009), research topics inherited features from the previous stage while also developing new ones. First, research topics in the second phase maintained the focus on medical data analysis and its related methodologies and technologies [30-32]. Medical image processing became a dependent hot spot, indicating that studies on medical image processing grew rapidly during this period [33-35]. Furthermore, as medical informatics became increasingly interdisciplinary, studies were no longer limited to analyzing data from medical institutions or medical systems. Web-based health information also attracted the attention of researchers, including studies on internet users’ information behavior (search [36], application [37], and evaluation [38] of web-based health information). Finally, the topics in stage 2 reflected the shift in emphasis from data to people, with more studies aimed at meeting patients’ health care needs [39-41] and improving users’ satisfaction [42,43].

In the third stage (2010-2020), medical data analysis remained one of the research hot spots. Derived from topics in stage 2, the purpose of medical informatics research always took user needs into account, including the needs of patients [44] and doctors [45]. Meanwhile, studies in this period also paid more attention to applying new emerging technologies in health data analysis, such as deep learning [46], blockchain [47], and artificial intelligence [48]. Furthermore, with the growing use of smartphones and wearables, a variety of health tools have enabled users to generate their own private health logs and manage their health conditions, such as weight control [49], chronic disease treatment [50], and mental health management [51]. Particularly during the COVID-19 pandemic, the use of digital health tools to provide health care and mental support for people became a significant issue [52]. However, as mobile health tools such as health apps have become widely used, researchers should pay attention to emerging problems such as the digital divide [53] and the patients’ privacy disclosure [54], especially older adults’ acceptance of information and communications technology [55].

On the basis of the results of research topic extraction in all corpora, we concluded that the focus of research in medical informatics could be divided into two aspects: data-centered studies and people-centered studies. In data-centered studies, medical records, medical images, and disease data were analyzed, which used mathematical methods and computing technologies to increase the efficiency and precision of data analysis. People-centered studies emphasized user needs and satisfaction, intending to improve health care service and health tool usability.

Furthermore, topic evolution patterns revealed that medical data analysis has always been a research hot spot since the beginning of medical informatics, particularly the methods and technologies used in data analysis. This is consistent with the results of previous studies [9,56]. The reason for this might be attributed to the development of emerging technologies, which prompted the exploration of data analysis methods. We could infer that future medical informatics research will continue to focus on the application of emerging technologies, such as deep learning, artificial intelligence, and blockchain, in medical data analysis. The topic evolution patterns also showed that people-centered topics arose in the second stage and were integrated with data-centered topics in the third stage. This tendency may be emphasized in future medical informatics studies. As mentioned previously, people-centered studies have considered user needs and satisfaction. It is possible that the usability of health tools such as health apps and wearables, as well as their effect on health behavior intervention, could be important issues for future research.

### Limitations

There are several limitations to this study. First, the Web of Science database did not index the abstracts of all papers, especially those in the early stage. As a result, we might have missed some topics in the research topic extraction. Second, we chose 27 representative journals in medical informatics without regard to the journals’ starting years. Journals that started in the earlier period would cover different topics from later ones, which might influence topic extraction results. Finally, while identifying the research stages, we only considered the annual cumulative number of research papers according to the literature growth curve of Price [10]. The journal amount, paper work, and web-based submission were also important indexes to consider when determining research stages.

### Comparison With Prior Work

We reviewed the development history of medical informatics from 1964 to 2020. Previous literature reviews have mostly focused on papers published within the last 10 to 20 years [3]. By contrast, our study attempted to provide a comprehensive review of medical informatics based on the results of a thorough survey.

In previous studies, research stages were usually divided intuitively based on the annual number of papers curve, with no quantitative model fitting [9]. In our study, we used the piecewise regression model to fit the curve of the annual cumulative number of papers to identify the research stages. We also used several mathematical models to fit curves in different stages to determine the literature growth features of each stage. We find that medical informatics is at a fast development stage, with an exponential increase in the literature. In fact, medical informatics has attracted research interest from various fields. Our findings are consistent with the current situation.

Previous studies that extracted research topics in medical informatics simply discussed and summarized the content of these topics [56]. In this study, we further divided the research topics into data- and people-centered topics. Furthermore, we found an integration tendency between these 2 types of topics according to their evolution patterns. However, previous studies have only emphasized the importance of medical data analysis [9].

### Conclusions

Our study offers a comprehensive understanding of research hot spots and their evolution patterns in medical informatics, and it could be helpful for predicting future research trends in this field. We found that medical informatics was in the fast development stage, with rapid growth in the literature. Medical data analysis has always been an important research topic since the birth of medical informatics to the current developmental stage. Many researchers are interested in data analysis methodologies and technologies, such as mathematical models and computer science technologies. In addition, the concentration of medical data has shifted from data to people. Recent studies have focused on improving medical systems and health tools, such as how to deliver better patient care and how to support users’ self-health management. We predicted that the application of emerging computer technologies in medical data analysis and the usability of mobile health tools would become a research hot spots in future medical informatics studies.

## Abbreviations

LDA: latent Dirichlet allocation
MeSH: Medical Subject Headings
